# The Reviews Are in: A Qualitative Content Analysis of Consumer Perspectives on Apps for Bipolar Disorder

**DOI:** 10.2196/jmir.7273

**Published:** 2017-04-07

**Authors:** Jennifer Nicholas, Andrea S Fogarty, Katherine Boydell, Helen Christensen

**Affiliations:** ^1^ Black Dog Institute University of New South Wales Sydney Australia; ^2^ School of Psychiatry Faculty of Medicine University of New South Wales Sydney Australia

**Keywords:** mobile applications, bipolar disorder, smartphone, telemedicine, qualitative research

## Abstract

**Background:**

The delivery of mobile health (mHealth) services is acceptable to mental health consumers. However, despite the benefits of accessibility, cost-effectiveness, anonymity, and ability to tailor content to individual needs, consumer engagement remains a hurdle for uptake and continued use. This may be unsurprising as few studies have examined app content from the consumer perspective or assessed consumer preferences for the content of apps for mental health management. An opportunity to examine consumer perspectives exists in using naturally generated data that is publically available in the Google Play and Apple app stores. Whereas commercial developers routinely use this data, to date there has been no in-depth evaluation within scientific research.

**Objective:**

The aim of our study was to explore what consumers consider useful content for mental health management apps, identify unmet needs, and understand user expectations of mental health apps within the context of apps for bipolar disorder.

**Methods:**

Publically available English language consumer reviews of 48 apps for bipolar disorder were used as data, providing a total of 2173 reviews. Review text was coded and analyzed using a team approach to qualitative content analysis. Results were presented in 2 forms: (1) a quantitative summary of the 9 major and minor themes and (2) a qualitative synthesis of key thematic findings.

**Results:**

The majority of reviews were for symptom monitoring apps (87.94%, 1911/2173). The qualitative content analysis revealed 5 main themes: (1) laudatory talk, comments regarding the app’s benefits including helpfulness and successful design features (74.00% of reviews, 1608/2173); (2) unfavorable feedback, negative reviews largely concerning unmet needs, privacy and technical issues, and potential dangers of app use (25.54%, 555/2173); (3) conceptions of community, referring to both communities of users with mental ill-health accessed via the app and a community created among app users and developers (24.25%, 527/2173); (4) wishlist features, app features requested by users (17.53%, 381/2173); and (5) apps and therapy, app use within clinical care (10.58%, 230/2173). Four minor themes were also identified: (1) app cost, (2) privacy and data security, (3) comparisons with traditional monitoring, and (4) evidence-based mHealth.

**Conclusions:**

Although mostly positive, the proportion of reviews containing wishlist requests indicates consumer needs are not adequately addressed by currently available disorder management apps. Consumers value content that is helpful, supportive, and easy to use, and they are integrating apps into their health management and clinical care without necessarily considering the evidence-base or clinical effectiveness of the tool. User expectations regarding developer responsiveness to their needs has implications for community-based participatory research and integrated knowledge translation. However, this expectation is incompatible with current mHealth funding structures.

## Introduction

The delivery of mobile health (mHealth) services is acceptable to mental health consumers [1,2]. Among the advantages of mHealth cited by consumers are convenience, the ability to identify triggers to mood states, and reducing isolation [1]. Furthermore, preliminary research indicates that the use of mHealth to facilitate symptom monitoring is acceptable to individuals with bipolar disorder [3]. Harnessing these qualities, smartphone apps have the potential to provide a platform for intervention and support for mental health conditions. Since the debut of the app store in 2008, the number of health and mental health apps available has risen exponentially [4]. However, no research has examined consumer perceived usefulness of apps for bipolar disorder management. This is despite the fact that apps may be uniquely suited to the condition; as active self-management is critical for bipolar disorder, with individuals reporting symptom monitoring and personal pattern identification as essential to disorder management [5,6].

A recent assessment of such mHealth tools developed for the disorder [7] found none demonstrated efficacy in reducing symptoms, preventing relapse, or facilitating disorder management. Moreover, few apps provided information grounded in evidence-based practice or adhered to clinical resources for disorder management. This has been further noted in mHealth research generally [8], with poor integration of evidence-based recommendations across apps for health [9,10] and mental health [11-13] conditions.

However, research beyond app evidence-base or efficacy is scant. Few studies have examined mental health apps from the consumer perspective or assessed consumer preferences regarding app content specifically for disorder management. This lack of attention to the consumer perspective may in part contribute to the modest app use statistics reported by several industry surveys. A US consumer survey found that 26% of health apps are abandoned after first use and overall 74% are discontinued within 10 uses [14], with engagement, finding a better app, and usability the main reasons for discontinuation. Similar research suggests only 5% of apps maintain continued use over a month after download [15].

Therefore, while delivery of resources and interventions using mobile technology employs the benefits of accessibility, cost-effectiveness, anonymity, and ability to tailor content to individual needs, consumer engagement remains a hurdle for uptake and continued use. An examination of consumer’s perspectives of apps is therefore a critical first step to improving the utility of such resources. The opportunity already exists to examine consumer perspectives using naturally generated data that is publically available in the Google Play and Apple app stores. App users provide information about features they value, need, and dislike when they choose to review the apps they use. App developers ensure they are meeting user expectations by examining review content [16] but to date there has been no comprehensive evaluation within scientific research.

In this paper, we perform a qualitative analysis of user reviews in order to (1) explore what app content users consider useful for bipolar disorder management and identify unmet needs, (2) understand user expectations of disorder management apps, (3) examine how apps for the disorder are being used, and (4) determine if users’ perspectives on current apps are consistent with scientific evaluation. Using qualitative methodology, we examine the main themes within user reviews and discuss the implications of these perspectives for app development and clinical practice.

## Methods

### Data Collection

The study used publicly available data. User review inclusion was restricted to the reviews of 82 apps previously investigated in a study of app content [7]. Detailed methods of app selection and assessment are outlined in Nicholas et al [7]; in brief, apps were identified by a search of the Google Play and Apple app stores using the following bipolar related search terms: bipolar, bipolar, “manic depression,” “mood swings,” “mania” and “mood,” cyclothymia, and cyclothymic. Apps with descriptions stating they were useful for the disorder, targeted at consumers or carers, and available in English were included in the study. All English language reviews submitted up to December 31, 2015 for the 82 apps included in Nicholas et al [7] were included in the analysis.

Android reviews were collected using a data extraction script, which included the text of the review, star rating given, and review date, as well as app details including name, package ID, and cost. Apple apps reviews were transcribed by hand, extracting the same information. Although reviewers were often logged into an Apple or Google Play account when reviewing an app, to maintain anonymity, no data about the review author was collected. For this reason, direct quotes within text and illustrative examples in Multimedia Appendix 1 have been minimally amended to preserve this anonymity, without changing the intent of the review. Reviews were transcribed or exported into Excel, cleaned and formatted, and then imported into the qualitative data management program NVivo11 (QSR International 2016) for analysis. The study received ethical approval from the UNSW Australia Human Research Ethics Advisory (Protocol number: HC14358).

### Data Analysis

#### App and Review Characteristics

Categorization of app function (eg, symptom monitoring) followed categories assigned to apps in Nicholas et al [7], which were determined by an extensive analysis of app content. Descriptive statistics were used to detail app price, number of reviewed apps, and number of reviews per app platform and app function. Average user-star rating by themes was calculated using NVivo11.

#### Qualitative Analysis

Data from the reviews were described using qualitative content analysis techniques [17,18], following a “conventional” approach, as is indicated for fields of research where existing theory is limited [17,18]. Content analysis was chosen for its strengths in systematically categorizing and summarizing large volumes of text-based data, and the ability to assist in interpreting patterns occurring in the text, with attention given to the context from which sample data is drawn [19]. We used a deliberately broad operationalization of qualitative content analysis, in line with previous research [17,20,21] which asserts that data derived from content analyses can be reported qualitatively without presenting full “counts” or statistical analysis. However, in recognition of more traditional and narrowly defined approaches to content analysis [22,23], we do present a brief quantitative summary of key features in the data.

Our approach to the analysis followed established coding techniques [24] over 3 broad phases: (1) immersion in the data, (2) reduction of the data through systematic coding and generation of themes, and (3) interpretation of the findings. Given the paucity of content analyses of consumer perspectives on app technology, the analysis used an inductive approach to developing a coding framework [25,26].

A preliminary sample of 10 pages of reviews was randomly selected to ensure adequate coverage across the dataset. Three coders (KB, JN, and AF) immersed themselves in the data by reading and rereading extracted reviews, followed by independently generating first stage concepts, or “main ideas” detected in the text. These main ideas were then compared among all 3 coders to check for similarities and differences before tentative agreement was reached regarding appropriate coding categories for the main ideas. These main ideas formed the basis for the quantitative component of the content analysis and are reported in the Results section as “major themes” if they were present in more than 10% of all reviews, or as “minor themes” where they were not [27]. Given the volume of data generated by over 2000 reviews, quantitative reporting of the content analysis was restricted to these major and minor themes.

Two coders (JN and AF) then selected another sample of 60 reviews to code in common, and 60 reviews to code independently. At this stage, the previously identified major and minor themes were tested and potential subthemes relevant to each of the major themes were identified. The team then met to discuss and agree upon a final coding framework to be applied to the remaining data. Detailed descriptions of the microlevel codes related to major, minor, and subthemes were generated in consultation with the study team. All remaining reviews were then evenly divided and coded line-by-line by 2 coders (JN and AF), who regularly checked in to resolve any coding disputes or discuss new codes detected in the data. Upon completion of coding, the coding framework was then applied to the original samples, which were then reintegrated into the dataset. Coders then met to refine reporting of results, including synthesizing information coded under each of the identified subthemes. Thus, results of the content analysis are reported as both a broad quantitative summary of the 9 major and minor themes [28] and a more descriptive qualitative summary of key findings in the subthemes [17].

### Research Rigor

We attended to research rigor in multiple ways, with specific emphasis on prolonged engagement with the data [29,30]. In our qualitative reporting, we established confirmability of results via consistent team debriefing related to the description and definition of themes and subthemes [30]. A second check involved both coders independently synthesizing key findings in the same subthemes, before comparing results. This process was repeated for three themes, before both coders developed an acceptable level of similarity in their approach. The remaining subthemes were then divided evenly between the 2 primary coders to report. For our quantitative reporting, intercoder reliability was assessed by using a random number generator to select 217 (10.00%, 217/2173) reviews to detect kappa with a null value of .40 at 90% power [31]. Each coder assessed the major and minor themes in each review and agreement between coders was calculated.

## Results

### Sample

**Figure 1:**
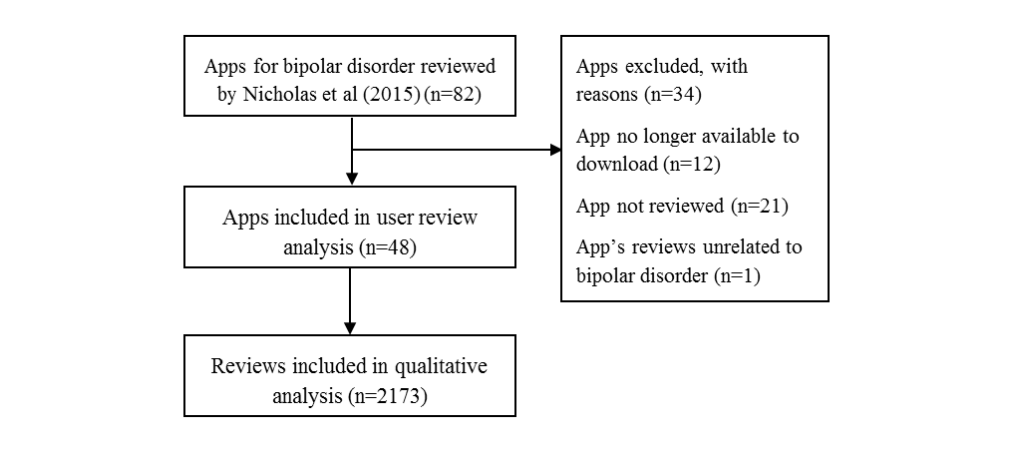
Prisma flow diagram of reviews included in the analysis.

Of the 82 eligible apps, consumer reviews of 48 apps were included in the analysis; 37 Android apps and 11 iOS (see Multimedia Appendix 2). Of the 34 excluded apps, 12 were no longer available for download, 21 had no reviews, and one app’s reviews were excluded as the app related to general health and reviews were unrelated to bipolar disorder. In total, users reviewed the 48 apps 2173 times (Figure 1). Reviews were submitted over a 10-year period, from July 13, 2005 to December 31, 2015, with the majority submitted after January 1, 2011.

### Description of Apps and Reviews

Thirty-six (75%, 36/48) of the included apps were free, and the average cost of paid apps was Aus $4.37. Just over half (52%, 25/48) were categorized as symptom monitoring, which accounted for 87.94% (1911/2173) of total reviews. Thirteen apps provided information about the disorder and had 122 reviews (5.61%, 122/2173). Very few apps and reviews related to screening, community support, or treatment provision (See Table 1). The majority of reviews (89.46%, 1944/2173) were for Android apps.

Five major and four minor (present in less than 10% of reviews) themes were identified in the data (Table 2). Table 2 also reports the percentage of reviews rated over 3 stars for each theme. Examples of subthemes identified within major themes and minor themes are displayed in Multimedia Appendix 1. Pertinent subthemes are discussed in further detail and were selected where results related to pragmatic implications for: clinical practice, design and development of app technology, and future research.

**Table 1:** Number of apps and reviews by app function.

App function	Number of apps with reviews (n=48)	Reviews (N=2173), n (%)	Average reviews per app
Symptom monitoring	25	1911 (87.94)	73.50
Information	13	122 (5.61)	9.38
Screening and assessment	6	73 (3.36)	12.17
Community support	3	63 (2.90)	21
Treatment	1	4 (0.18)	4

**Table 2:** Prevalence of major and minor themes identified in the data (categories not exclusive; N=2173).

Themes		Reviews, n (%)	Reviews above 3 star rating, n (%)
**Major themes**			
	1. Laudatory talk	1608 (74.00)	1501 (93.35)
	2. Unfavorable feedback	555 (25.54)	150 (27.03)
	3. Community	527 (24.25)	411 (77.99)
	4. Wishlist	381 (17.53)	295 (77.43)
	5. Apps and therapy	230 (10.58)	214 (93.04)
**Minor themes**			
	1. App cost	149 (6.86)	78 (52.35)
	2. Privacy and data security	138 (6.35)	77 (55.80)
	3. Comparisons with traditional monitoring	26 (1.20)	25 (96.15)
	4. Evidence-based mHealth	5 (0.23)	5 (100.00)

### Research Rigor

Intercoder reliability for the 9 major and minor themes was strong, with almost perfect agreement for the themes laudatory talk (kappa=.838, *P*<.001, 95% CI 0.736-0.940), unfavorable feedback (kappa=.882, *P*<.001, 95% CI 0.808-0.956), and app cost (kappa=.85, *P*<.001, 95% CI 0.683-1.017). Substantial intercoder agreement was observed for 3 major themes: wishlist (kappa=.741, *P*<.001, 95% CI 0.629-0.853), apps and therapy (kappa=.714, *P*<.001, 95% CI 0.528-0.900), and privacy and data security (kappa=.701, *P*<.001, 95% CI 0.593-0.989). There were moderate levels of intercoder agreement for community (kappa=.493, *P*<.001, 95% CI 0.350-0.636). Perfect agreement was observed for comparison with traditional monitoring (kappa=1, *P*<.001), and both coders agreed that none of the randomly selected reviews contained any reference to the evidence-based mHealth theme.

### Major Theme 1: Laudatory Talk

Almost two thirds of reviews featured positive commentary about the app. However, positive reviews often included a contrasting statement, most commonly a request for additional features, for example, *“I love this, but...I want the ability to type longer notes.”* Positive reviews were often general in nature and did not provide specific details regarding which aspects of the app were highly valued, though “ease of use” heavily featured. Two main laudatory subthemes are discussed: (1.1) helpfulness and (1.2) successful design.

#### Subtheme 1.1: Helpfulness

Many positive reviews indicated that the app was helpful, but did not specify exactly how. Of the reviews that did elaborate, the app assisted in two interrelated areas—keeping track of and gaining insight into moods. Whereas mood was the main symptom monitored, reviewers used the apps to monitor a range of factors including sleep, medication, and episode triggers. Gaining insight into mood was often attributed to the app’s ability to provide an increased understanding of affect changes, including identification of mood triggers or establishing duration of and variations in mood. For some, this insight was otherwise occluded by mood state, for example, *“It can be hard to see when you’re in it.”* A few users spoke about gaining control of their mood through app use and identified that understanding and insight brought power and self-efficacy, for example, “*This helps me predict and deal with mood changes*. *I can prepare for down moods and reassure myself that these feelings are normal for me at the time and that it will pass.”*

#### Subtheme 1.2: Successful Design

Many reviews were complimentary of the app’s features or overall design. A commonly described positive app feature was ease of use—simple, easy, intuitive, and quick to learn, for example, *“I like this app. It’s easy to use, and that means I’m more likely to use it.”* When specific app features were praised, these were similar to features requested within major theme 4, wishlist (shown in Table 3). Most commonly praised features were symptom monitoring options, provision of graphs and analysis, reminders, and the app’s interface.

### Major Theme 2: Unfavorable Feedback

Negative comments were present in just over a quarter of reviews and mostly concerned overall app design and functionality. Here, general comments were less common, with negative reviews concentrating on 3 key areas: (2.1) app content or features not meeting user needs; (2.2) deal breakers, features that prompted users to cease using an app, or technical issues that interfered with app use; and less commonly, (2.3) the potential for apps to precipitate or worsen distress.

#### Subtheme 2.1: Features Not Meeting User Needs

A recurring complaint concerned the lack of fit between user experience of symptoms and available features. This was particularly evident where reviewers self-identified as having “rapid-cycling bipolar disorder” and reported mood fluctuations throughout the day, not adequately captured by once-daily monitoring options. Besides wanting to track more frequently, many also wanted to select from a more extensive range of feelings, symptoms, or behaviors, for example, *“There is a very very small mood selection. Just moods like ‘happy, sad, depressed’ nothing like ‘disappointed, irritated, proud’ which are important.”* Similarly, though not as common, were complaints about the inability to customize monitoring scales, for example, “*Grr. The scale goes 0-10, but instead of 0 being very anxious and 10 being very calm, its 0-10 anxiety...with the middle marked as ‘normal’.”*

#### Subtheme 2.2: “Deal Breakers” and Technical Issues

The aspects of apps considered deal-breakers were repeatedly mentioned. The majority of “deal breakers” involved concerns about privacy or technology failure. Users also discontinued use when available options were inadequate, the app used too much data or processing power, or when tasks were too burdensome, for example *, “Too complicated to do when sad.”* Occasionally, reviews referred to inaccurate or unclear app content, lack of instructions, difficulty learning phone gestures (eg, tap and hold), or advertising as reasons for discontinuing use.

Similarly, technical issues that interfered with app use featured prominently in negative reviews. For example, inactive export features; apps that crashed, lost data, froze, did not load content, or would not download; apps that were slow, unresponsive, or unavailable offline; apps without developer support; and apps that were buggy or “glitchy.”

#### Subtheme 2.3: Potential to Precipitate Distress

Less commonly, reviewers were disparaging of apps they thought could potentially cause or exacerbate distress via provision of poor information or failure to consider vulnerable users. Some noted that app developers lacked necessary clinical expertise, for example, *“This person is a quack not a dr...unsafe and irresponsible.”* Some did not specify the perceived danger but noted the app had the capacity to harm help-seekers by attracting them with promises that the app failed to deliver. Less commonly, users described feeling more distressed or frustrated after engaging with the app, for example*, "Of course it’s because of mood swings that one gets a mood app. So imagine what happens when the first time trying to open the app it doesn’t work!!!*

### Major Theme 3: Community

About a quarter of all reviews referred to feeling part of a “community” that extended well beyond the apps. Generally, community referred to (3.1) a wide interactive community of app users (actual or potential) and developers or (3.2) a Web-based community accessed via the app.

#### Subtheme 3.1: An Interactive Community of App Users and Developers

Within the app use community, reviews often directly addressed app developers, most commonly to thank, encourage, or make requests. At times, requests were “incentivized” with reviewers promising improved star ratings or donations in return for added features or fixes. There was also an emphasis on developer responsiveness, with reviewers pleased when concerns raised with developers were addressed. Less frequently, reviewers expressed confusion over the apps intended function, or questioned the developers’ expertise in creating a mental health app.

The community of other app users was referenced during interactions with developers, to support and backup requests, for example, “*My manic brothers understand the need to write paragraphs at times. Please fix this.”* This sometimes extended to referring to another user’s review as evidence of a shared need. The community was not restricted to those with bipolar disorder, extending to any potential users, with many reviews including opinions about the apps usefulness to subgroups of individuals with mental ill-health. Although they were mostly positive about who would benefit, some reviews served as a warning to others about inadequate privacy or potentially harmful content.

#### Subtheme 3.2: App-Accessed Web-Based Communities

Reviews related to Web-based communities typically referred to their benefits. Some reviewers reported that they gained help from interactions with other users, viewing it as a resource to improve their mood when depressed, for example, *“It helps when you’re feeling low.”* The major benefit discussed was a normalizing experience, with reviewers gaining a sense of connection and understanding through communicating with others with mental ill-health. This understanding through shared experience was often expressed as something that was difficult to access in other aspects of life, for example, “*It helps get your problems out if you can’t tell anyone else.”* The direct or immediate support gained through Web-based communities was often reciprocated, with users providing encouragement to others.

However, not all found community interaction positive. Some expressed an unwillingness to have strangers privy to their experience of the disorder, for example, “*I really don’t want the world to know how I feel.”* The few negative reviews among those eager to engage with a community concerned the unresponsiveness, coldness, or immaturity of other users.

### Major Theme 4: Wishlist

Approximately one in five reviews included a wishlist suggestion, that is, features reviewers thought would improve their app experience or health management. Reviewers made both direct requests for app changes and requested the ability to customize, with direct requests more common. Wishlist requests are displayed in Table 3; requests for tracking options were the most prevalent. Of the 34 additional symptoms requested for tracking, sleep was the most common.

**Table 3:** Requested features of apps for bipolar disorder.

Request frequency	Feature requested
Highly requested features	Customizable symptom tracking
	Multiple entries per day
	Export data
	Graph changes
	Additional tracking categories; sleep, medication, diet, mood triggers, exercise, anxiety, substance use
	Reminders
	Edit or delete inputs
	Longer section for notes
	Mood scale range or options
Minimally requested features	Personal identification number or password security
	Information backup
	User interface changes
	Social interaction capability
	Between app integration
	Sync across devices

### Major Theme 5: Apps and Therapy

About one in ten reviews mentioned using apps in partnership with clinical services. Generally, these were (5.1) perspectives of app users and a minority of health care practitioners who saw the potential clinical utility of apps or (5.2) perceived therapeutic effects of app use.

#### Subtheme 5.1: Using Apps With Clinical Care

Comments were contributed, in the main, by app users, followed by a minority of health care practitioners (HCPs). HCPs indicated they were either recommending apps to clients or evaluating apps with a view to making recommendations in future. For HCPs, apps were another tool that could help their clients develop insights into their moods, triggers, and behaviors; provide feedback; and facilitate between session symptom management.

Consumers agreed that apps were a useful adjunct to clinical care, though sometimes for different reasons. Whereas users also mentioned “insight,” they reported that apps could help their HCPs gain greater insight into their experience of the disorder. For example, apps could relay their experience with mood swings, for example, “*This app has helped me and my clinician both better understand my mood changes,”* responses to medication and general progress to guide treatment decisions based on a more “accurate” record of their symptoms. The concept of accuracy was recurring, with apps taking the onus off memory and in turn improving communication with their HCP, for example, “*I can’t remember what brings on my bipolar episodes and this allows me to accurately tell my psychiatrist how my moods have been.”* To facilitate this communication, many commented on the capacity (existing or desired) for apps to export data to show HCPs. An increased likelihood of participating in between session activities was also expressed, for example, *“Can now do my therapy homework.”* Finally, there was a sense that in sharing apps with their HCP, they might be helping others in similar situations.

Rarely, reviews identified the ability of apps to support family care. These reviewers used apps to monitor behavior or symptoms in their partners, children, or other family members not restricted to bipolar disorder.

#### Subtheme 5.2: Therapeutic Effects of App Use

A few users indicated the app itself had a therapeutic effect, positively affecting mood or well-being. Being able to see mood changes was reported as a motivator to do well, particularly if this data was visible to or shared with others. A few reviewers developed a therapeutic alliance or partnership with the app. Typically, these reviews alluded to working on mental health “together” with some reference to receiving support from the app, for example, *“We have made it through thick and thin together.”*

### Minor Theme 1: App Cost

The minority of reviews that referenced app cost were varied in nature. Reviews indicated a willingness to pay for one-off donations, “pro” versions, removal of advertising, and overwhelmingly, a good product. “Good products” were easy to use, aesthetically pleasing, worked seamlessly, integrated with other software, or synced to other devices, and importantly, contained features deemed “useful” or “helpful.” Whereas many that had purchased apps indicated that it was worth it, those who disagreed often used strong language, for example, “*I feel like I’ve been swindled.”* Cost influenced app expectations with reviewers using price as a guide, for example, *“For the price it really needs improvement”* or *“Good for a freebie.”* In general, negative reviews related to app prices were largely complaints about paying for features or services not received (eg, poor tech support or apps that crashed or froze).

### Minor Theme 2: Privacy and Data Security

Similarly, a minority of reviews related to app privacy or data security. Data privacy and security was generally conceived of in terms of handset access, with most comments related to using a personal identification number, login, or password to secure access to the app and data stored within it. Generally, people supported these approaches. Other comments concerned cyber access, permissions requested by the app in the terms of service use, with main concerns regarding data storage practices or data sharing with third parties, for example, “*I began to feel that this app was a useless little spybot.”*

However, most comments about privacy were generated in response to one particular app, which was designed so that the mood journal was public by default and made private by upgrade purchase.That is, user entries could be seen and commented on by other app users, as part of a Web-based community, unless users paid Aus $1. This approach uncovered conflicting perspectives. Contrasting views surrounded which should be default, public, or private entries, with some stating that privacy should be default, for example, *“Already a stranger commented on my mood...it’s no-one’s business! I’m uninstalling this app—imagine if my stalker found it!”* Others were open to sharing personal information and highlighted benefits of the community created (eg, finding support). Linking privacy to app cost was also contentious. Some users reported that privacy was a right, and therefore, not for sale, whereas others felt that privacy was worth paying for and, in this case, was inexpensive. For those who objected to linking privacy and price, the language used was notably strong, for example, manipulative, cruel, terrifying, offensive, greedy, unethical, extortion, black mail, morally wrong, and intolerable.

### Minor Theme 3: Comparisons With Traditional Monitoring

A small minority of reviews addressed the benefits of app-based tools over traditional paper and pencil resources. Benefits included increased access, ease, and convenience, leading to increased reliability, for example, *“Loving the simplicity compared to paper methods.”* Increased reliability was also linked to an app’s ability to remind users to complete monitoring, for example, *“I’ve set a reminder so I’ll definitely fill it in daily.”* Although collectively positive, a few reviews stated a preference for paper and pencil tools as they were seen as more advanced, or more secure, for example, *“I trust paper and pen a little more.”*

### Minor Theme 4: Evidence-Based mHealth

Minimal reviews commented on the evidence-base or scientific quality of an app. Those that did referred to research or scientific involvement or reference to well-known tools in development, or health professional endorsement, as indications of app quality.

## Discussion

### Principal Findings

This is one of the first explorations of consumer perspectives on currently available mHealth tools for mental health using publically available review data. The mixed qualitative method employed here builds on previous approaches [32] and allowed for both a quantitative summary of the main themes and fine-gained exploration of important subthemes. Our data show that user feedback on mental health apps could generally be summarized by 9 themes, with variation in their distribution across the reviews. Reviews varied in length and specificity, with many providing rich, detailed text with important consumer perspectives on the potential for apps to be helpful tools for mental health management.

The proportion of reviews containing positive and negative themes was similar to previous results in general apps [27]. When compared with reviews of apps that reduce harmful drinking [32], reviews of apps for bipolar disorder were more favorable. The main content of negative reviews reported here also supports previous literature citing functionality issues, lack of features, and crashing as the most common complaints [32,33]. However, reviews were rarely restricted to one theme, indicating that consumer needs and expectations about apps are complex and multidimensional, with no currently available app providing a satisfactory balance.

The predominance of contrasting phrases and “wishlist” requests within the reviews indicate that consumer needs are not adequately addressed by currently available apps for the disorder, even given the generally positive reviews. Many of the most requested features, for example, monitoring additions and reminders, were highlighted previously as areas in which apps failed to conform to evidence-based practice [7]. A comprehensive review of the features and scientific quality of the same apps noted that 65% had inadequate mood scales and reminder features were often absent or not functioning as intended [7]. These examples highlight failure to translate scientific best practice and consumer considerations into satisfactory and functional apps for self-management and likely reflect the high proportion of private, individual, or corporate developers [7]. However, user requests also extended beyond features present in clinically used self-management resources, indicating a need to balance user preference and clinical relevance.

Despite these unmet needs, reviewers were largely positive about the apps, valuing content that was supportive, helpful, and easy to use. However, sustaining such helpfulness and engagement throughout an episodic condition represents a specific challenge. The prevalence of reviews that referred to community demonstrates the importance of connectedness and social support in managing mental health, as well as the potential for Web-based communities to provide that support. Although previous research has been inconclusive about the benefits of Web-based communities for health problems [34,35], our results indicate that consumers perceive benefits, even when the community had not been sought. This concords with research in which consumers emphasize the importance of support obtained through app communities [32]. Consumers reported that app communities were normalizing, supportive, and mutually beneficial, something that has been established in other health problems [36]. Users who gained understanding and support from community members often spoke of providing similar support to other users. Indeed, reciprocity has been noted as an important aspect of the social nature of self-management and thus, app communities have the potential to provide this support to individuals who may otherwise not receive it [37]. Furthermore, as mental health related stigma is well documented and interferes with people’s willingness to access support and mental health services [38,39], apps with associated Web-based communities may in part remove this barrier to support by being anonymous, ubiquitous, and inexpensive.

Although the overwhelming majority of reviews about community discussed the understanding supportive environment created, the potential for negative experiences requires acknowledgment. Many expressed unwillingness to discuss their mental health with others, emphasizing that community elements should be upfront and engagement optional. Indeed a review of Web-based health communities posited that willing engaged participants were necessary for communities to be beneficial [35]. Active participants may also reduce incidences of disappointment and distress expressed after not receiving support when reaching out. Although, negative effects of studies involving Web-based communities were not reported [35], such lack of support when solicited could potentially enhance feelings of stigma and isolation commonly experienced by those with mental health disorders [40]. Given the potential for supporting those in need, but also possible negative effects, it is crucial that this balance is considered during app development and that apps are derived, where possible, from a clear evidence-base and best clinical practice.

Although this need for established efficacy or evidence-informed content in mHealth tools for mental health is widely acknowledged by mHealth researchers and clinicians [7,41-43], discussion of scientific quality was strikingly absent from consumer reviews. This could represent genuine disinterest in the scientific basis of mental health apps, or could reflect a lack of health app regulation knowledge among consumers. With most apps classified as health and fitness or medical [7], there may be an assumption that they are on the app store because they “work.” In this case, a reviewer would discuss scientific quality only if an app obviously violated that assumption. Due to the growing number of apps for mental health [4], it is imperative that consumer “app-literacy,” knowledge regarding evidence and data privacy of apps, is understood. This understanding is vital for successful knowledge translation of evidence-based mHealth tools and requires that future research investigate consumer knowledge about app use for mental health. There is also a clear role for researchers to develop better communication and advocacy regarding clinically effective apps built on robust scientific data.

A further user expectation elucidated in the analysis was that developers would address their needs and perspectives. This was an implication of the wider community of other app users (active or potential) and developers that was identified around apps. Previous research reinforces the importance of developer responsiveness, reporting that developers who respond to user reviews receive a 1-point increase in star rating, approximately a third of the time [44]. This conception of community has important implications for community-based participatory research and knowledge translation. Whereas currently this interplay between users and developers is occurring after an app’s deployment, acknowledgment by the user base that they have useful knowledge regarding app features and disorder management, highlights the potential for engaging consumers throughout the development process. Consumer participation in design has obvious advantages for the resulting app’s function and uptake and use by the target community [45,46]. Furthermore, consumer involvement in the delivery of services can effectively support recovery, with related benefits in consumer empowerment, social inclusion, satisfaction with services provided, and well-being [47,48]. Capitalizing on this potential in Web-based service delivery is an important avenue for future research, with consumer involvement actively recommended [47]. Such recommendations have recently been echoed by The International Society for Bipolar Disorders working group on consumer research involvement [49].

In keeping with the concept of meaningful partnerships, several reviews contained information related to routine clinical management, as frequently, disorder specific apps were not used independently. Reviewers used apps to inform and support their relationship with their HCP, with emphases on improved recall and feeling better understood during clinical appointments. Many reported that sharing app data with their HCP helped to represent their lived experience more fully, potentially promoting greater therapeutic alliance [50]. Clearly, apps for mental health are not viewed as replacements for clinical care, but rather as useful adjuncts to treatment.

Whereas the potential of technology for scaling mental health interventions and delivering mental health services is recognized for Web-based mental health resources [51], the recency of the field and lack of an established evidence base mean such initiatives have not extended to mHealth. Our results show that apps are already used by consumers looking for tools to manage their health, which supports anecdotal evidence that consumers are driving the introduction of apps into clinical practice. Little research has explored HCPs’ attitudes to the use of apps in clinical care, yet current data indicate that some consumers expect HCPs to be open to app use and some HCPs are already attempting to determine the best available tools. Given the current lack of evidence-based mHealth resources, there is a clear need to support both consumers and HCPs with an interest in using apps distinguish those of high quality.

### Limitations

This study is not without limitations. The data used were publically available existing text, rather than data gathered through more exploratory methods, using more refined and/or explicit questions. It is possible that there may be salient themes for app development and disorder management that are elided by the data gathering techniques used here, or that important consumer attitudes have not been adequately captured. Similarly, we cannot be clear about the needs or preferences of consumers in the community who have no experience using apps to manage their mental health. However, given richness of the data contained in these reviews, the large number of reviews recorded across multiple apps spanning more than five years, we can be reasonably confident the findings reported here represent real concerns held by consumers in the community. Future research will need to further clarify and confirm that the themes reported resonate with a wider community of consumers and explore the needs of those who have not previously engaged with mHealth technologies.

Reviews of apps for bipolar disorder were restricted to apps reported in Nicholas et al [7]. However, this methodology had the clear advantage that the function and quality of apps reviewed were known and consumer perspectives could be compared with quality assessment. Nevertheless, we acknowledge that in the time since this review, more apps for the condition are likely to have been made available, therefore potentially limiting study comprehensiveness. Finally, as the app marketplace is dynamic and transforming [52], the apps, their features, and opinions of consumers are subject to change.

### Conclusions

This paper provides a unique perspective on consumer attitudes and expectations toward mental health apps, and hence, represents an important contribution to the knowledge base in the expanding research area of mHealth. Consumers value content that is helpful, supportive, and easy to use, and they are integrating apps into their health management without necessarily considering evidence-base or the clinical effectiveness of the tool. Such consumer insights are vital to our ability to be competitive in the unregulated app store environment but also reveal the need to balance user preferences and clinical relevance. Indeed, integrated knowledge translation strategies involving consumers in all stages of mHealth research may be critical to ensuring uptake and continued use, and results indicate such research strategies would be acceptable to consumers.

The expectation of developer responsiveness has implications for research app development and mHealth resource funding. Currently funding awards do not account for resource sustainability costs, a critical facet of existing and competing in the dynamic app marketplace. To realize the potential of mHealth to support self-management, increase access to care, and provide mental health resources, a change in app development practices and funding structures for such resources is required.

## Appendix

Multimedia Appendix 1 Examples of subthemes and minor themes identified.

Multimedia Appendix 2 List of apps included in the analysis.

## Abbreviations

mHealth: mobile health
HCP: health care practitioner
