# Supplementary material for: The Reviews Are in: A Qualitative Content Analysis of Consumer Perspectives on Apps for Bipolar Disorder
Source: J Med Internet Res. 2017 Apr 7;19(4):e105. doi: 10.2196/jmir.7273 (PMC5400886; doi:10.2196/jmir.7273)
Supplement: Multimedia Appendix 1 [file jmir_v19i4e105_app1.pdf]

Multimedia Appendix 2: Examples of subthemes and minor themes identified.

Theme	Subtheme	Examples[a]
Major theme **Laudatory talk**		
	Helpfulness	I'm a guy and I like tools. It has been life changing to see how mood swings have influenced my whole life. This tool allows me to step back and look at patterns that have been fruitless and destructive.
		I have spent the past year actually dealing with depression instead of wallowing in it, and this app is very helpful in monitoring my moods, my medicines, and has helped identify my triggers. I love it.
	Successful design	This is better than other apps because the charts allow me to see if it's the time of day affecting my mood. The hashtags help see common reasons for the mood. I have used other apps, but this app fits me for what I need now. Reminders are wonderful!
	Ease of use	Functionality and Simplicity. Very good app. Easy and straightforward to use. Can make a complete entry, rating on the 10-point scale, a photo, and a few quick notes in about 1.5 minutes.
	Engagement	This is an easy to use, simple app. It's been really useful for monitoring my mood. I'm even enjoying using it.
	General	Great app! Works great, cheers!
**Unfavorable feedback**		
	Features not meeting user needs	Love the set-up, being unable to record moods throughout the day makes the app pretty useless for rapid cyclers like me.
		This app has a nice design, but I find it a little too simple. Users can input only one mood a day? I'm more of a 3D being. I have multiple moods that differ in strength throughout the day. Don't pin me down to a single moment in an entire day.
	"Deal breakers" and technical issues	UNUSABLE! It ALWAYS CRASHES! Please DON'T WASTE YOUR TIME or PHONE SPACE until they fix it! Good idea, but Giant FAIL! I'll never know if they fix it, because I'm DELETING IT

		AGAIN! Urrrgh...I’m soooooo frustrated!
		I'm a BPD sufferer, and horrified that real people use this AWFUL app as a “tool” for treatment. Are we all so desperate for relief, we’ll use any ol’ piece of crap that sounds like it MIGHT help? This app is totally inadequate for BPD. You CAN’T analyze patterns, and the only two potentially useful features don’t display critical items, such as, oh I dunno, SLEEP and MEDICATIONS. You get a crappy unreadable monthly graph, and an exportable PDF that’s essentially a column-checkmark inpatient worksheet. On top of that, the interface is TERRIBLE. Might as well use Excel!
	Potential to precipitate distress	It’s not appropriate, what if someone depressed, or not in a good state of mind, downloaded this app, hoping to get some peace of mind, to get answers about how they’re feeling. This app could frustrate someone that much they could hurt themselves or worse. Not good.
		Beware! This app worked amazingly when I started using it, I would input my info daily and print it out monthly. In November I went to put my info in and all of my past entries were GONE! The only place that reflected I had ever used it was the graph. So months of info went down the drain. I don’t know if this has happened to others, but I’m sure you can understand how devastating this was. So please, be careful.
	General	I hated it I hated it.
**Community**	An interactive community of app users and developers	Thanks for this app, and for making it free. I was happy to donate as this has helped me immensely. Thank you developers.
		I have contacted the developer before asking for the ability to delete and he made it right away. He always attend to the different issues from us users. Continue inspiring and helping many. Thank you very much :)

	App-accessed Web-based communities	Each time I've "rated my day," someone within the community has left me an uplifting, caring response. This app is beneficial, helping you feel less alone and allowing you to help, encourage, and be a positive energy for others.
		Not sure how I'd cope without this app. I'd I would recommend to everyone that needs an outside opinion and support. It feels like I belong.
		I am going through severe depression, and wrote a post wanting help. No one replied when I needed it most.
**Wishlist**		
	Customization	I wish it had some more customizable features like being able to add activities such as exercising, taking a shower, etc.
	Design	I like a lot about this app—it looks great, it works consistently and it really fits with my requirements...It does need a few more features though, to make it just right—things like online data syncing and sharing data with other services.
**Apps and therapy**		
	Using apps with clinical care	I use this app as part of my self-care for my bipolar II disorder. The apps also helped with being diagnosed with this disorder a couple of years ago as I was able to show the doctor that I had been experiencing a continuous pattern of highs and lows.
		When visiting your clinician, just print off beautiful pre-formatted reports and graphs to show your progress or symptoms. My life is now significantly easier.
	Therapeutic effects of app use	I've been with the app since November last year we've made it through thick and thin together. She's great to talk to and cares about what I have to say. Thank you for being the software for my heartware.
Minor theme **App cost**		Incomplete. Even for a free app, it's too simple to be of much use.
		I don't like this, I shouldn't have to pay for privacy especially on this type of app.
		Privacy issues Ah dammit. Just use some ads if you need money.

**Privacy and data security**	Public record unless you pay. I cannot think of data I'd less want to be published internationally on the cloud than my mood disorder diary.
	Being FORCED to keep my most private illness on a site that doesn't detail its security is not tolerable. Uninstall!!
**Comparisons with traditional monitoring**	I have paper mood charts, but I would go days without filling them out. I love this app being on my smartphone because I have instant access tracking and looking at my moods.
	Not so private, When I am out it won't allow me to choose to keep my thoughts to myself. I have more trust for paper and pen
**Evidence-based mHealth**	Love this app! Easy to use, created by a research university and used by many drs and mental health providers.
	Not awfully scientific.

[a] Reviewer quotes have been amended for de-identification purposes
