# Supplementary material for: The Reviews Are in: A Qualitative Content Analysis of Consumer Perspectives on Apps for Bipolar Disorder
Source: J Med Internet Res. 2017 Apr 7;19(4):e105. doi: 10.2196/jmir.7273 (PMC5400886; doi:10.2196/jmir.7273)
Supplement: Multimedia Appendix 2 [file jmir_v19i4e105_app2.pdf]

Multimedia Appendix 2: List of apps included in the analysis.

App ID	Platform	Title	Developer	Function	Price	Star rating[a]	Number of reviews[a]
1	Android	Recovery International	Brian Corson	Information	$0.00	4.3	6
6	Android	eMoods Bipolar Mood Tracker	Yottaram LLC	Symptom monitoring	$0.00	4.0	318
7	Android	Bipolar Treatment	Karl Evans	Information	$0.00	3.5	8
8	Android	Bipolar Test	Consurgo	Screening and assessment	$0.00	3.1	24
9	Android	Bipolar Tracker	William Alexander	Screening and assessment	$0.00	2.8	20
10	Android	Bipolar Connect	Alliance Health Network	Community support	$0.00	2.8	36
11	Android	Bipolar	Kindle Trove Apps	Information	$0.00	3.5	6
12	Android	Mood Chart Bipolar Depression	Monitor My Health, LLC	Symptom monitoring	$0.00	1.6	8
15	Android	Bipolar Disorder Uncovered	KoolAppz	Information	$1.29	5.0	1
31	Android	Psych Central	Liviant LLC	Information	$0.00	4.4	41
32	Android	Free Mood Tracker	Invicta Trading and Promotion HB	Symptom monitoring	$0.00	2.0	11
44	Android	Health Tips	JD Star	Information	$0.00	3.1	8
49	Android	Personal Psychology Tests	Darren Gates	Screening and assessment	$0.99	4.2	3
56	Android	iMoodJournal	Inexika Inc	Symptom monitoring	$0.99	3.3	225
58	Android	Personal Progress Tracker	SoundMindz.Org	Symptom monitoring	$0.00	4.6	43
60	Android	Moodlytics, Smart Mood Tracker	AnantApps by Avinashi	Symptom monitoring	$0.00	3.9	262
62	Android	Moodtrack Diary: Mood Tracker	Matthew Windwer	Symptom monitoring	$0.00	4.0	604

63	Android	1-800-therapist	1-800-therapist	Information	$0.00	4.2	16
64	Android	Primary School Assessments	Darren Gates	Screening and assessment	$0.99	3.9	1
65	Android	Smiley Calendar	3 ACORN Technologies, LLC	Symptom monitoring	$0.00	5.0	2
69	Android	Bipol-App	Healthcare Learning Smile-o	Symptom monitoring	$0.00	3.5	10
70	Android	In Flow - Mood Diary	AITA LTD	Symptom monitoring	$0.00	1.8	137
72	Android	Preschooler Assessments	Darren Gates	Screening and assessment	$0.99	4.0	1
76	Android	RxWiki	RxWiki, Inc	Information	$0.00	5.0	13
78	Android	MHF	Together For Change	Community support	$0.00	4.0	12
80	Android	How Are You - Mood Tracker	Quantum Lab Co.	Symptom monitoring	$13.82	3.3	19
81	Android	Yoga for you	Yashendu Goswami	Information	$0.00	3.9	10
82	Android	dailyRx	dailyRx, Inc	Information	$0.00	4.3	8
83	Android	WhatsMyM3	M3 Information	Screening and assessment	$0.00	3.6	24
90	Android	Depression 101 by WAGmob	WAGmob (Simple 'n Easy)	Information	$2.14	3.8	1
95	Android	Mood O Scope : Mood Tracker	XLabz Technologies Pvt Ltd	Symptom monitoring	$0.00	5.0	9
106	Android	MoodiModo BETA - Mood Tracker	The Abolitionist Project	Symptom monitoring	$0.00	3.2	27
114	Android	Mood Tracker By: CTHF	Cheryl T. Herman Foundation	Symptom monitoring	$0.00	4.3	14
122	Android	Mental Health At Work	eMETA Learning Solution	Information	$0.00	1.9	3
138	Android	Control	mhapps development	Information	$0.00	5.0	1
167	Android	Daily Mood	Kaizen Dev	Symptom monitoring	$0.00	4.7	10
177	Android	Mood diary - depression	Julia Bechmann	Symptom	$2.96	4.0	2

				monitoring			
328	iOS	iMoodJournal -mood journal	Inexika Inc.	Symptom monitoring	$2.49	4.6	98
329	iOS	Bipolar Disorder Connect	Alliance Health Networks, Inc	Community support	$0.00	3.7	15
330	iOS	Optimism	Optimism Apps Pty Ltd	Symptom monitoring	$0.00	4.6	64
333	iOS	Moody Me - Mood Diary and Tracker	Medhelp	Symptom monitoring	$0.00	2.6	10
339	iOS	How Are You - depression, bipolar and mood	Quantum Lab Co.	Symptom monitoring	$16.99	3.0	1
345	iOS	In Flow - Mood and Emotion Diary	AITA LIMITED	Symptom monitoring	$0.00	4.4	26
351	iOS	MoodLog	Markus Schopfer	Symptom monitoring	$0.00	5.0	3
353	iOS	Bipol-App	Healthcare Learning Company Ltd	Symptom monitoring	$0.00	2.3	4
357	iOS	Better Mood Tracker - A Quantified Self Research Tool	Alexander Stone	Symptom monitoring	$0.00	2.5	2
358	iOS	iCouch CBT	iCouch Inc.	Treatment	$4.99	4.5	4
376	iOS	Personal Mood Chart Journal	Monitor My Health, LLC	Symptom monitoring	$3.79	3.0	2

[a] at time of data acquisition (12/12/2015)
